# Altered Long Noncoding RNA Expression Profile in Multiple Myeloma Patients with Bisphosphonate-Induced Osteonecrosis of the Jaw

**DOI:** 10.1155/2020/9879876

**Published:** 2020-07-02

**Authors:** Alessandro Allegra, Manuela Mania, Angela D'Ascola, Giacomo Oteri, Enrico Nastro Siniscalchi, Angela Avenoso, Vanessa Innao, Michele Scuruchi, Andrea Gaetano Allegra, Caterina Musolino, Salvatore Campo

**Affiliations:** ^1^Division of Haematology, Department of Human Pathology in Adulthood and Childhood “Gaetano Barresi”, University of Messina, 98125 Messina, Italy; ^2^Department of Biochemical and Dental Sciences and Morphofunctional Images, University of Messina, Messina, Italy; ^3^Department of Clinical and Experimental Medicine, University of Messina, Italy

## Abstract

Bisphosphonates (BPs) are inhibitors of osteoclast-mediated bone resorption used for the treatment of multiple myeloma (MM) patients with osteolytic lesions. Bisphosphonate-induced osteonecrosis of the jaw (BONJ) is an infrequent drug-caused adverse event of these agents. Long noncoding RNAs (lncRNAs) are a set of more than 200 base pairs, noncoding RNA molecules, which are critical posttranscriptional regulators of gene expression. Our study was aimed at evaluating 17 lncRNAs, whose targets were previously validated as key elements in MM, bone metabolism, and angiogenesis in MM subjects without BONJ (MM group), in MM subjects with BONJ (BONJ group), and a group of healthy controls (CTRL group). Our results demonstrated a different lncRNA profile in BONJ patients compared to MM patients and controls. Two lncRNAs (DANCR and MALAT1) were both downregulated compared to controls and MM, twelve (HOTAIR, MEG3, TP73-AS1, HOTTIP, HIF1A-AS2, MANTIS, CTD-2201E18, CTD1-2003C8, R-471B22, RP1-43E13, RP11-553L6.5, and RP1-286D6) were overexpressed in MM with BONJ, and one (H19) was upregulated compared with only MM. Two lncRNAs (JHDMD1 and MTMR9LP) had higher expression, but these differences were not statistically significant. The examined lncRNAs target several genes and metabolic pathways. An altered lncRNA signature could contribute to the onset of BONJ or have a protective action. Targeting these lncRNAs could offer a possibility for the prevention or therapy of BONJ.

## 1. Introduction

Long noncoding (lnc) RNAs are a set of noncoding RNAs longer than 200 base pairs [1]. lncRNA biogenesis is similar to that of protein-coding RNAs and mRNA, since most of them have a poly-A tail; however, they cannot be translated into proteins [2]. For this reason, lncRNAs were believed to be “transcriptional noise” with no biological actions [3]; however, whole-genome transcriptomic investigation demonstrated that they are implicated in several biological functions [4].

To date, 15,778 human lncRNAs have been recognized [5, 6], although only a little part of these is typified. lncRNAs comprise enhancer RNAs, intergenic transcripts, and snoRNA host [7]. They have been discovered in almost every cell type and act as central controllers of numerous cellular activities, comprising cell proliferation, cellular architecture, cell cycle progression, nuclear-cytoplasmic passage, and transcriptional and posttranscriptional control. Moreover, they act on the epigenetic regulation of gene expression [8–12].

lncRNAs have different mechanisms of action. They can fold into a tertiary structure and offer support for the creation of a quaternary structure for proteins [13]. Moreover, they regulate the gene expression at the posttranscriptional level by influencing the stability of mRNAs, changing the translation effectiveness of target mRNAs, and determining augmented mRNA expression [10].

Tumours are the consequence of genomic instability due to an alteration of the systems regulating cell proliferation, survival, and apoptosis. Several lncRNAs have been recognized as relevant components in tumour genomics, and decreased or increased expression of lncRNAs in tumour cells is connected to better or poor prognosis [14].

Multiple myeloma (MM) is a malignant neoplasm of plasma cells conducting bone lesions and marrow failure. Bioinformatic analysis recognized more than 3000 dysregulated lncRNAs in MM patients [15], while 176 lncRNAs were identified as biomarkers for the prognosis of the MM subjects [16, 17].

Moreover, alteration of lncRNAs could be crucial in the onset and progression of the disease. It has been discovered lncRNA KIAA0495 showed a gradual downregulation from healthy controls to MGUS to symptomatic MM.

Finally, lncRNAs could play an essential role in bone metabolism and perhaps in MM bone disease. Recent experimentations have evaluated the action of lncRNAs during osteogenic lineage commitment or osteocyte terminal differentiation [18–21]. For example, lncRNA-1 displayed augmented expression during osteogenesis. Moreover, knockdown of lncRNA-1 expression in primary animal preosteoblasts was found to block osteogenic differentiation, as demonstrated by a decreased transcription of the Sp7 and Runx2/p57 bone master genes [22]. Recent findings have also shown that lncRNAs have a crucial action in angiogenesis, by modulating several mediators, such as vascular endothelial growth factor (VEGF) [23].

Bisphosphonates (BPs) are drugs employed for the therapy of bone lesions, including those connected to MM. Generally, they are a well-tolerated drug; however, several reports have described osteonecrosis of the jaw (ONJ) as a potentially serious adverse effect associated with the use of these drugs [24].

The pathophysiology of BONJ (bisphosphonate-induced osteonecrosis of the jaw) has not been completely clarified. Possible factors comprise the block of osteoclastic bone resorption and remodelling, inhibition of angiogenesis, or repeated microtrauma. The other causes include alteration of humoral and cell-mediated immunity and BP toxicity in soft tissues. Moreover, infections and inflammation are central elements of BONJ, namely, persistent exposed bone in the jaw [25–28].

In previous works, we have demonstrated the presence of a modified microRNA signature in the peripheral lymphoid compartment of MM subjects and MM patients with BONJ [29, 30].

Our research was aimed at evaluating 17 lncRNAs, whose targets were previously validated and reported as key elements in MM, bone metabolism, and angiogenesis in MM patients without BONJ (hereafter identified as the MM group), MM patients with BONJ (hereafter identified as the BONJ group), and healthy controls (hereafter identified as the CTRL group).

## 2. Materials and Methods

### 2.1. Samples

The study was in accordance with the ethical standards of the responsible committee on human experimentation (institutional and national) and with the Helsinki Declaration of 1975, as revised in 2008. The Local Ethics Committee approved this study protocol before the initiation of any study-related procedures (Protocol No. 36/18 of 07 May 2018—resolution No. 887).

After every subject was informed about the research and informed consent was signed, venous blood samples were collected in tubes containing a heparin anticoagulant from 8 healthy subjects (CTRL group, 4 men and 4 women, median age 57 ± 10 years), from 8 MM patients without BONJ (MM group, 5 men and 3 women, median age 58 ± 8 years), and 8 MM patients with BONJ (BONJ group, 3 men and 5 women, median age 60 ± 9 years). The mandible was more frequently implicated (five patients) than the maxilla (three patients).

MM and BONJ patients had been treated with BPs. The duration of therapy with zoledronic acid was higher than 1 year in all patients.

According to the Durie–Salmon staging system, in the MM group, five patients were MM disease stage II and three patients were disease stage III. Median plasmocytosis of bone marrow was 65% (range 51–89%). The paraprotein class was immunoglobulin G (IgG) in all patients.

As regards the BONJ group, according to the Durie–Salmon staging system, four patients were MM disease stage II and four patients were disease stage III. Median plasmocytosis of bone marrow was 74% (range 56–95%). The paraprotein class was immunoglobulin G (IgG) in all patients.

The patients' characteristics are summarized in Table 1.

The blood samples were diluted (1 : 3) in PBS, mixed gently, and layered onto equal amounts of Lymphoprep (Cederline, Canada) for density centrifugation at 800g for 30′ at room temperature, to allow stratification of the cells on the medium. Then, the buffy coat was collected with a Pasteur pipette, transferred onto a clean tube, washed 3 times with PBS, and centrifuged at 600g for 10′, discarding the supernatant each time.

### 2.2. RNA Isolation and cDNA Synthesis

Total RNA was extracted from a lymphomonocyte pellet using the TRIzol reagent (Life Technologies, USA), according to the manufacturer's instructions. Total RNA was quantified at 260 nm (40 ng/ml RNA = 1.0 OD) using a spectrophotometer (BioMate 3, Thermo Electron Corporation, Marietta, OH, USA); its purity was assessed by the ratio of readings at 260 nm and 280 nm. The integrity of total RNA was checked by denaturing agarose gel electrophoresis and fluorochromatization with ethidium bromide.

Total RNA was transcribed into cDNA through a high-capacity cDNA reverse transcription kit (Applied Biosystems, CA, USA), according to the manufacturer's recommendations.

### 2.3. Selection of lncRNAs and RT-qPCR

lncRNAs were chosen based on their role as key factors in bone homeostasis (HOTAIR, MALAT1, MEG3, TP73-AS1, HOTTIP, and DANCR) and numerous types of human cancer, including myeloma (HOTAIR, MALAT1, MEG3, H19, MANTIS, RP1-286D6, MTMR9LP, RP1-43E13, RP11-553L6.5, CTD-2201E18, CTD1-2003C8, and R-471B22) and angiogenesis (HIF1A-AS2, MANTIS, and JHDMD1). The expression profile was measured by real-time qPCR using a 7500 Real-Time PCR System (Applied Biosystems, CA, USA). Reactions were performed using the PowerUp SYBR Green Master Mix (Applied Biosystems, CA, USA) to test a total of 17 lncRNAs and *β*-actin, used as an endogenous control for the subsequent data normalization. Primer sequences were designed in-house for each lncRNA, as reported in Table 2. A denaturation cycle was added at the end of all reactions to evaluate the specificity of each result.

The RT-qPCR results were analyzed using the 2^-*ΔΔ*Ct^ method for relative quantification, using the average of *Δ*Ct values from the control subjects as a calibrator. The results are expressed according to the 2^-*ΔΔ*Ct^ calculation as fold change relative to controls.

### 2.4. Statistical Analysis

All data were analyzed by one-way analysis of variance (ANOVA) followed by the Student–Newman–Keuls test, using PRISM software (version 5.0; GraphPad Software, CA, USA). The statistical significance was set at *p* < 0.05, and data are expressed as the mean ± S.D. values. All assays were repeated three times to ensure reproducibility.

## 3. Results

### 3.1. Detection of Significantly Dysregulated lncRNAs in ONJ

**Figure 1:**
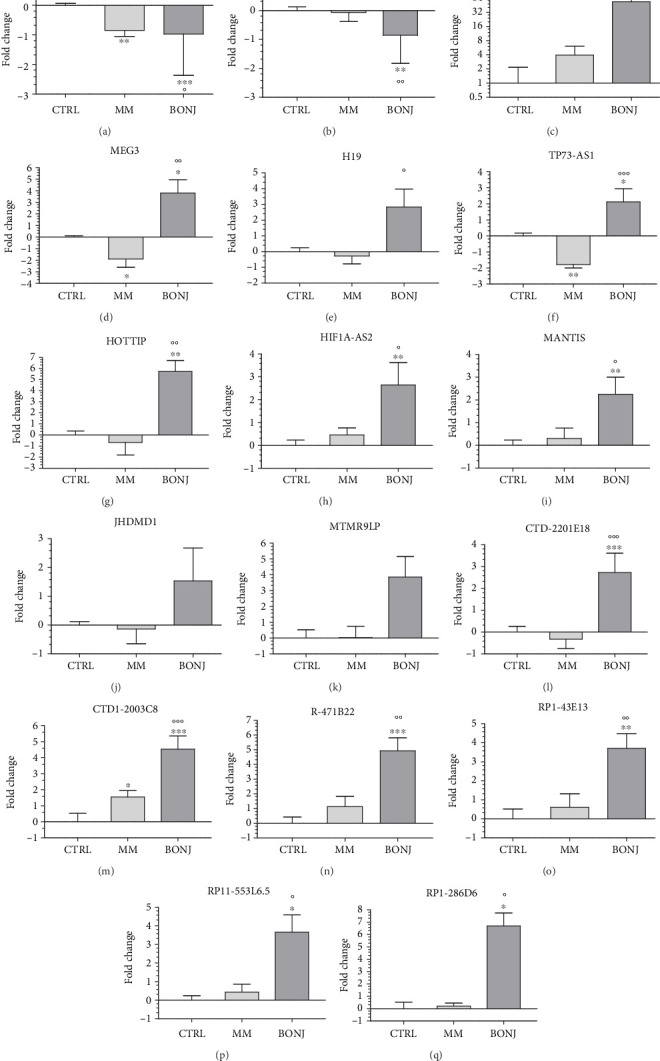
RT-qPCR analysis of DANCR (a), MALAT1 (b), HOTAIR (c), MEG3 (d), H19 (e), TP73-AS1 (f), HOTTIP (g), HIF1A-AS2 (h), MANTIS (i), JHDMD1 (j), MTMR9LP (k), CTD-2201E18 (l), CTD1-2003C8 (m), R-471B22 (n), RP1-43E13 (o), RP11-553L6.5 (p), and RP1-286D6 (q) lncRNA expression levels in multiple myeloma patients (MM) without and with bisphosphonate-induced osteonecrosis of the jaw (BONJ). Values are expressed in a log_2_ scale as the fold change with respect to the healthy controls. ^∗^*p* < 0.05, ^∗∗^*p* < 0.01, and ^∗∗∗^*p* < 0.001 vs. controls; °*p* < 0.05, °°*p* < 0.01, and °°°*p* < 0.001 vs. multiple myeloma patients (MM).

We performed an analysis on 17 lncRNAs involved in bone homeostasis, cancer, and angiogenesis. Our data revealed that 15 lncRNAs were significantly differentially expressed in BONJ patients compared with both CTRL and MM subjects (Figure 1).

In particular, in BONJ patients, two lncRNAs were downregulated compared to CTRL and MM (DANCR and MALAT1, see Figures 1(a) and 1(b)), twelve were overexpressed compared to either CTRL or MM (HOTAIR in Figure 1(c), MEG3 in Figure 1(d), TP73-AS1 in Figure 1(f), HOTTIP in Figure 1(g), HIF1A-AS2 in Figure 1(h), MANTIS in Figure 1(i), CTD-2201E18 in Figure 1(l), CTD1-2003C8 in Figure 1(m), R-471B22 in Figure 1(n), RP1-43E13 in Figure 1(o), RP11-553L6.5 in Figure 1(p), and RP1-286D6 in Figure 1(q)), and one was upregulated compared with MM only (H19, see Figure 1(e)). The last two lncRNAs JHDMD1 and MTMR9LP (see Figures 1(j) and 1(k)) had higher expression levels in BONJ subjects compared to either MM or CTRL subjects, but all these variations were not statistically significant.

## 4. Discussion

The possibility to use new drugs, such as immunomodulatory drugs, proteasome inhibitors, monoclonal antibodies, and inhibitors of heat shock proteins; vaccine therapy; or adoptive immunotherapy has drastically bettered the clinical outcome of MM subjects [31–36].

However, the use of polychemotherapy for disease treatment and its complications expose MM patients to the onset of even more serious side effects such as BONJ. For this reason, the identification of novel therapeutic targets seems imperative.

Recently, Wang et al. described the lncRNA expression profile of bone marrow mesenchymal stem cells (BMSCs) from subjects with steroid-induced osteonecrosis of the femoral head (SONFH). 1878 lncRNAs were upregulated and 1842 lncRNAs were reduced in SONFH patients, and several lncRNAs of these were involved in osteogenic differentiation [37].

In our study, we established a different lncRNA signature for MM patients with BONJ compared to MM patients without BONJ and healthy controls. These lncRNAs target several genes and biological pathways involved in bone formation, osteogenic differentiation, osteoblastic differentiation, angiogenesis, and bone repair in the tooth extraction socket. Their alteration could, therefore, constitute a contributing factor in the pathogenesis of BONJ.

For instance, although the action carried out by BPs on bone metabolism and, in particular, on osteoclasts and osteoblasts is certainly not the sole pathogenetic factor, it assumes great importance in the onset of the disease. This is also demonstrated by the fact that numerous biomarkers of bone metabolism are altered in patients with BONJ. For instance, PTH level is statistically higher and TSH, Vit-D, osteocalcin, and NTX levels are statistically lower compared to the control group [38, 39].

Bisphosphonates can affect osteoclast-mediated bone resorption in a variety of ways, including effects on osteoclast recruitment, differentiation, and resorption, and they can induce apoptosis [40]. Compared to osteoclasts, the scientific literature regarding the effect of bisphosphonates on osteoblasts is less conclusive. The conflicting apoptotic and antiapoptotic effects could be explained by the different bisphosphonates studied and concentrations used [41]. Mounting evidence suggests that cells of the osteoblast lineage are affected directly by bisphosphonates in a dose-dependent manner that contributes to the development of BONJ [42].

In our study, we have shown that some of the long noncoding RNAs examined, capable of intervening in osteogenesis and the activity of osteoclasts, are differently expressed in patients with BONJ.

Regarding the influence of DANCR (Differentiation Antagonizing Nonprotein Coding RNA) on bone metabolism, it decreases osteogenic differentiation blocking the p38MAPK pathway [43] and inhibiting the Wnt/*β*-catenin pathway [44]. Moreover, this lncRNA decreases the expression of the transcription factor FOXO1, which in turn increases osteoblast differentiation and reduces osteoblast proliferation [45, 46]. A study aimed at evaluating *DANCR* expression in human periodontal ligament stem cells demonstrated that downregulation of *DANCR* was crucial for osteogenesis [47]. Our results show reduced expression of DANCR in MM patients with BONJ. Lower lncRNA levels in MM patients with BONJ could correlate with the inhibition in osteoblast differentiation, which in turn affects the jaws during the development of lesions.

The downregulation of MALAT1 (Metastasis-Associated Lung Adenocarcinoma Transcript 1) could have a similar meaning. It controls concentrations of integrins, such as ITGB1, which perform a relevant action in osteoclast genesis and cytoskeletal structure [48]. MALAT1 regulates miR-124 which in turn negatively controls bone formations and osteogenic differentiation by working with Dlx transcription factors [49].

Our data revealed significant downexpression of MALAT1 compared to both the controls and MM patients, probably related to increased osteoclast genesis associated with bone lesions.

Through different mechanisms, also the upregulation of some lncRNAs could have a negative action on osteogenesis.

HOTAIR (HOX Transcript Antisense RNA) can inhibit osteogenic differentiation. It was observed that the expression of HOTAIR was greater in patients with nontraumatic osteonecrosis of the femoral head (ONFH) compared with osteoarthritis samples. The concentration of osteogenic differentiation biomarkers, comprising COL1A1 and RUNX2 mRNA levels, was increased by si-HOTAIR [50]. Moreover, HOTAIR is mechanoresponsive and therefore may have an action in mechanically controlled calcification.

HOTAIR revealed higher expression in MM patients with BONJ compared to both controls and MM patients. These results were in line with the findings found in nontraumatic osteonecrosis of the femur, indicating that this lncRNA could negatively control osteogenic proliferation and differentiation [50].

A partially different effect could instead be exercised by the upregulation of H19. It is a 2.3 kb lncRNA that can control numerous components with a regulatory action on osteogenesis [51]. H19 can operate as a “sponge” to reduce the action of microRNAs that modulate the expression of proosteogenic proteins, including the Wnt/*β*-catenin pathway and its target genes.

Our data revealed an increase in H19 expression levels compared with both the controls and MM patients, and this, in turn, could negatively influence osteogenesis.

Among the potential mechanisms capable of inducing BONJ, an essential role is attributed to the possibility that bisphosphonates may have an antiangiogenetic action capable of delaying wound healing and/or affecting microinfarction in bone and/or soft tissues [52].

In this context, a different production pattern of lncRNAs could also play an essential role, and the overexpression of lncRNAs such as MEG3 (Maternally Expressed 3) or Jumonji could result in an important suppression of vascularization in patients with BONJ.

The MEG3 lncRNA gene can modify the expression of angiogenesis-promoting genes [53] while, in MEG3-knockout mice, augmented expression of VEGF pathway genes and microvessel density were reported [54, 55]. Our results show that MEG3 is upregulated in MM patients with BONJ compared to controls and MM. It is conceivable that its action on angiogenesis could play a role in the onset of avascular necrosis typical of BONJ.

The overexpression of Jumonji C could have an analogous meaning. It can reduce angiogenesis [56], and its increase could contribute to the onset of microinfarcts typical of BONJ.

Regarding the particular meaning that the overexpression of lncRNA HOTTIP (HOXA transcript at the distal tip) could assume, some findings suggest that systemic bisphosphonate treatment influences the activity of chondrocytes [57]. HOTTIP has been reported to interact with WDR5, forming a complex with TWIST1 [58]. In cranial bones, Twist1 induces a reduction of chondrogenesis via *β*-catenin [59]. Moreover, this lncRNA is an enhancer that controls the activity of 5′*HOXA* genes to regulate the elongation of skeletal components via epigenetic mechanisms.

Data obtained from the present study revealed that HOTTIP was significantly overexpressed in MM patients with BONJ compared to either the controls or MM, indicating a possible inhibiting effect on chondrogenesis and osteogenesis.

Finally, it is worth noting that a change in the expression of some lncRNAs may have a protective action against the onset of BONJ.

TP73 antisense 1 (alias PDAM/TP73-AS1) may control apoptosis-modulating p53-dependent antiapoptotic genes. In bone metabolism, HMGB1 is correlated with angiogenesis and bone remodelling by osteoclast and osteoblast activation. It stimulates bone healing in the tooth extraction socket [60]. In our study, TP73-AS1 was upregulated in MM patients with ONJ compared to both controls and MM. High TP73-AS1 levels may contribute to bone healing. Therefore, an increase in lncRNA could have a protective meaning for the onset of BONJ.

Similarly, the overexpression of other lncRNAs could positively modify both angiogenesis and osteogenesis.

MANTIS is expressed in endothelial cells, and a reduction of MANTIS expression causes altered endothelial sprouting and decreases endothelial migration [61, 62]. Current literature has demonstrated that bisphosphonates have a strong negative influence on angiogenesis, revascularization, and microvessel sprouting. MANTIS is upregulated in MM patients with BONJ compared to either controls or MM. It is conceivable that this increment is due to a tissue effort to allow bone regeneration.

HIF1A-AS2 lncRNA eases the increase in HIF-1*α* by sponging to miR-153-3p, also promoting angiogenesis. Furthermore, HIF1A-AS2 increases osteogenic differentiation of adipose-derived stem cells via miR-665/IL-6 through the PI-3K/Akt signalling pathway, augments concentrations of osteoblast markers osteocalcin, Runx2, and osterix, and increases ALP activity [63]. Since our results showed an increase in HIF1A-AS2 in MM patients with ONJ compared with both healthy controls and MM patients, it may have a protective action against the progression of osteonecrosis.

Finally, in different studies, MTMR9LP, RP1-286D6 (GenBank: AL365330.16), RP1-43E13.2 (GenBank: AL357564.17), RP11-553L6.5 (GenBank: AC093010.7), CTD-2201E18 (GenBank: AC008875.9), CTD1-2003C8 (GenBank: AC069360.7), and R-471B22 (GenBank: AL512791.3) were identified as prognostic markers, since they positively correlated with the survival of MM patients [16, 64]. Our data showed that all these lncRNAs (except MTMR9LP) were significantly overexpressed in MM patients with BONJ compared to either MM patients or controls.

These lncRNAs are implicated in several pathways connected to MM progression. However, the real action of these lncRNAs on the onset of BONJ is not well known [65] and needs further investigation to unravel its molecular mechanisms. Moreover, their different expression in MM patients with or without BONJ compared to controls could also be due to the bidirectional influence of the tumour microenvironment (i.e., osteoblasts, osteoclasts, endothelial cells, and bone marrow stromal cells) on MM cells and vice versa, which is crucial for malignant plasma cell proliferation and the development of drug resistance [66]. In fact, recent research has identified molecular interactions between myeloma cells and the bone marrow microenvironment, which can also be disruptive to the environment that supports them, thus leading to myeloma development and associated complications, such as bone lesions due to osteolysis [67]. Also, at the same time, a better understanding of the signalling pathways involved in myeloma has led to the development of new targeted therapies, which are improving the quality of life for patients and significantly extending median patient survival [68].

## 5. Conclusions

An increasing body of evidence shows that lncRNAs can be targeted to treat cancers. Moreover, many studies have also demonstrated that regulatory components controlling lncRNA modification can also be targeted for tumour treatment [69].

Due to their specificity, lncRNAs may be better therapeutic targets than current protein-coding genes for several tumours. However, at present, little is known about the activity of the majority of lncRNAs in the onset, progression, and diffusion of cancer. Therefore, their employment as therapeutic targets requires a great quantity of investigation. Nevertheless, lncRNAs represent an incalculable prospective as powerful controlling molecules, and they could be used not only as a biomarker but also as therapeutic targets [70]. The expression of lncRNAs can be blocked using RNAi tools, which have been created to knockdown lncRNAs. A different approach could be represented by antisense oligonucleotides or small-molecule inhibitors. Finally, gene therapy can be tried for the release of favourable cancer-suppressive lncRNAs [71, 72].

The introduction of the modulation of lncRNA activity into clinical practice to treat BONJ is certainly still a premature hypothesis. Albeit our study is preliminary and has some limitations regarding the number of subjects studied (too small to draw definitive conclusions), future interesting studies are needed to analyze lncRNA expression levels in the osteoclasts affected by bisphosphonates, thus leading to BONJ. Finally, it could be also appropriate to evaluate the targets of the analyzed lncRNAs and check the involved pathways (PI3K/Akt, mTOR signalling, and p38/MAPK) to further highlight the importance of the lncRNA expression profile in BONJ.

In the end, studying the effects of lncRNAs on different aspects of MM and BONJ pathophysiology could help us to better understand the intimate mechanisms that regulate the onset and progression of these diseases and make their treatment easier.

## Figures and Tables

**Table 1:** Number of controls and patients and clinical features of the examined disease.

Group	No. of patients (males, females)	Age (years)	Area with BONJ	BP treatment (yes/no (duration))	Durie–Salmon disease stage (no. of patients)	Median plasmocytosis (range)	Ig class
CTRL	8 (4 M, 4 F)	57 ± 10	N/A	No	N/A	N/A	N/A
MM	8 (5 M, 3 F)	58 ± 8	N/A	Yes (>1 year)	II (5) III (3)	65% (51–89%)	IgG
BONJ	8 (3 M, 5 F)	60 ± 9	Mandible (5 patients) Maxilla (3 patients)	Yes (>1 year)	II (4) III (4)	74% (56–95%)	IgG

CTRL: control group; MM: multiple myeloma patients without bisphosphonate-induced osteonecrosis of the jaw; MM+BONJ: multiple myeloma patients with bisphosphonate-induced osteonecrosis of the jaw; BP: bisphosphonates.

**Table 2:** Primers used for RT-qPCR analysis of lncRNAs.

Gene	Forward primer 5′-3′	Reverse primer 5′-3′
*β*-Actin	TTGTTACAGGAAGTCCCTTGCC	ATGCTATCACCTCCCCTGTGT
CTD-2201E18	TCTATGCTCCTCCTGCTTACG	GGCGGTTCCTCTTCTGATGTA
CTD1-2003C8	GGAGGCTGGAGGAAGAGATAAG	GTATGGAGAAGCTGCAGGCA
DANCR	GCCACTATGTAGCGGGTTTC	CGTAAGAGACGAACTCCTGGA
H19	CCAGAACCCACAACATGAAAG	TCACCTTCCAGAGCCGATT
HIF1A-AS2	ATGAGTTGGAGGTGTTGAAGC	TTTGCTCTTTGTGGTTGGATCT
HOTAIR T1	GCACTCACAGACAGAGGTTTA	CTCTGTACTCCCGTTCCCTAGA
HOTTIP T2	CAGGTTTGTCTGAGAGGGATG	CGCCACATTTAAGGAGCAAAG
JHDMD1	CCACAACACCCAAATAAGGACT	GGAGGGATTCACAGGCATTT
MALAT1	GGAAAGCGAGTGGTTGGTAA	ATCCCTTTACACCTCAGTACGA
MANTIS	CTGCTTACTCCTGTCAACCAA	TTTCTATTACCGATGCCTTTCTGT
MEG3	GTCTTCCTTCCTCACCTCCAA	TGCTTCCATCCGCAGTTCTT (A)
MTMR9LP	GTGACAGGAAGGGAGAAGACAG	CAAGGAGCCAGTGCTTAGAATAG
R-471B22	ACAGAGACAGAGAACCAACCA	GAGGCAATCAGAACACCGAAT
RP1-286D6	TGAGCTGAGCAGTGTCCTTA	CCTCCTGTTCGTGAGTCTCT
RP1-43E13	AAGCAGGTGGTAGCGACTTG	TTGGCTCTGGAGACGGAAT
RP11-553L6.5	GCAGTTTCCATTTCCCAGTG	TGCCTCTCCCTCTTTCCAAA
TP73-AS1 T1	CGGGATCTCACAGGCTTTAAA	ATCCCCGGCTCCCATCTA

## Data Availability

Readers can access the data supporting the conclusions of the study directly in the manuscript; no unavailable data are present elsewhere. Further details are available upon request.
